# The Effects of Progesterone on Oocyte Maturation and Embryo Development

**Published:** 2013-07-31

**Authors:** Mojdeh Salehnia, Saeed Zavareh

**Affiliations:** 1 Department of Anatomy, Tarbiat Modares University, Tehran, Iran; 2 Department of Cellular and Molecular Biology, School of Biology, Damghan University, Damghan, Iran

**Keywords:** Progesterone, Oocyte, Embryo, *in vitro* Maturation

## Abstract

Oocyte maturation and embryo development are controlled by intra-ovarian factors such as steroid hormones. Progesterone (P4) exists in the follicular fluid that contributes to normal mammalian ovarian function and has several critical functions during embryo development and implantation, including endometrial receptivity, embryonic survival during gestation and transformation of the endometrial stromal cells to decidual cells.

It is well known that the physiological effects of P4 during the pre-implantation stages of some mammal’s embryos are mediated by P4 receptors and their gene expression is determined. The effects of P4 on oocytes and embryo development have been assessed by some investigations, with contradictory results. P4, a dominant steroid in follicular fluid at approximately 18 hours after the luteinizing hormone (LH ) surge may have a critical role in maturation of oocytes at the germinal stage. However, it has been shown that different concentrations of P4 could not improve *in vitro* maturation rates of germinal vesicles (GV) in cumulus oocyte complexes (COCs) and cumulus denuded oocytes (CDOs). Culture media supplemented with P4 significantly improved mouse embryo development. In addition, an in vivo experimental design has shown high blastocyst survival and implantation rates in P4-treated mice.

In this review we explain some of the findings that pertain to the effects of P4 on oocyte maturation and embryo development both *in vitro* and in vivo.

## Introduction

Oocyte maturation and embryo development are controlled by steroid hormones as well as intra- ovarian factors such as cytokines and growth factors (1-4). In vivo, oocyte maturation takes place in the presence of follicular fluid which is composed of plasma exudates and secretions of follicular cells. With each follicular developmental stage, the steroid contents of follicular fluids change and the ratio of progesterone (P4) to estradiol (E2) is related to the maturation stage of the oocytes (5-7).

During folliculogenesis the oocyte gains its developmental competence in a gradual and sequential manner, after which it becomes a fully mature oocyte with the capability to become fertilized and develop into a high quality embryo (8).

The process of mammalian oocyte meiosis takes place in several steps. Initiation of the first meiotic division leads to primary oocytes that occur in the fetal development period or around the time of birth. Oocytes progress through zygotene, pachytene and early diplotene stages but arrest at the dictyate stage of prophase I. At puberty the first meiotic division is completed by a surge of luteinizing hormone during the menstrual cycle; the second meiotic arrest of the oocytes occurs at ovulation. Resumption of the second meiotic division occurs after penetration of the sperm (9).

### P4 structure and production

P4 is a cholesterol-derived, phylogenetically old steroid hormone (10). It is synthesized during the steroid hormone metabolizing pathways from androgens, estrogens, and glucocorticoids within several cell types such as the corpus luteum, placenta and adrenal gland. In addition it is produced from a plant steroid precursor, diosgenin (11, 12).

Within the ovary, cholesterol is converted by an enzyme to pregnenolone, another precursor steroid, after which it can follow one of two pathways (Δ4 or Δ5). In the Δ4 path way pregnenolone is converted to P4. P4 not only serves as aprecursor for other steroids, but enters the female’s blood and acts as a hormone on target tissue (13).

The level of plasma P4 varies with sex and reproductive age. P4 is mainly bound to albumin, however it has an affinity to bind to corticosteroidbinding globulin. In the normal menstrual cycle, its levels rise during the follicular phase and reach a maximum level after ovulation. Its half-life in serum is about 5 minutes (10-12, 14).

### P4 function

P4 is an intra follicular steroid that plays critical roles in ovulation, implantation and maintenance of pregnancy (15, 16). P4 is the dominant content of follicular fluid steroids in mammalian preovulatory follicles, which are temporary and elevated at 18 hours after the luteinizing hormone (LH) surge (17).

P4 was initially studied as a contraceptive agent by inhibition of the luteinizing hormone surge and ovulation (18). However, it has a critical function in pregnancy maintenance and in the regulation of different biological functions in the ovarian tissue and feto-maternal unit such as resumption of meiosis, fertilization, embryonic development and implantation (19-21).

Clinically, it can be used in the female reproductive system as luteal support during *in vitro* fertilization (IVF) (22), hormone replacement therapy for older women (23), and as treatment for endometriosis and polycystic ovarian syndrome in younger women (24). In addition, P4 has immunological functions for the maintenance of a fetomaternal allograft (19).

Some investigations showed that P4 administration for luteal support improved uterine receptivity at the ultra structure levels (25-27) and enhanced the implantation rate in mice (28).

In our experiments we used ovariectomized animal models and injected exogenous hormones to evaluate the effects of P4 on endometrial morphology and gene expression. Our observations showed that exogenous P4 administration affected expression of endometrial integrin molecules (29, 30).

### P4 receptors in oocytes and embryos

The biological actions of P4 are mediated by three genomic isoforms of P4 receptors (PR), PRA, PR-B and PR-C, in addition tothree non-genomic isoforms, alpha, beta, and gamma (31, 32). Although PR-A and PR-B arise from a single gene, PR-A is a more important repressor than PR-B (33). The PR-C isoform is the shortest isoform. PR-C does not have transcriptional activity, however it has a role in decidual cells during late pregnancy (31, 34).

Both PR-A and PR-B are expressed in preovulatory folliclegranulosa cells (35-37). The membrane PR or non-genomic PGR are particularly notable as promoters of oocyte meiosis. They are expressed in neural, kidney, and intestinal tissues in addition to the reproductive tract (32).

Mice that lack PR-A and PR-B isoforms are infertile as a result of ovulation failure (38). It has been suggested that induction of PR isoforms in cumulus cells and their binding to P4 appear to affect follicular growth, oocyte maturation, and embryo development (39, 40).

Although PR was identified in granulosa cells, there was no evidence of this receptor on the oocyte (41, 42) with the exception of one report which observed the PR receptor in Xenopus laevis oocytes (43). Canine oocytes have been shown to express estrogen receptors during the estrous cycle, however, there is a lack of PR expression in all phases (41).

PR membrane component 1 (PGRMC1) is another potential mediator of P4 action (44). Possibly PGRMC1 mediatesanti-apoptoticactions of P4 (45). Western blot analysis has demonstrated the presence of PGRMC1in bovine germinal vesicle (GV) and metaphase II (MII) oocytes (46) as well as rat (44), and human oocytes (47). PGRMC1 expression is not only associated with male and female pronuclear formation, it is also highly expressed in blastocysts (46).

Despite high level expression of PGRMC1, little is known about its role on oocyte function, however it may be directly involved in the regulation of meiotic maturation (48).

Despite the lack of PGR ( PR) expression in the oocyte, both P4 and estrogen receptor mRNA and proteins have been detected in mature cumulus oocyte complexes (COCs) and embryonic cells from several mammalian species (49-55).

Aparicio and colleagues (39) have shown decreased bovine embryo development as a result of blocking genomic PR and non-genomic PR alpha activity. This result indicates that P4 intracellular signaling is mediated by its interaction with nuclear and membrane PRs and is also important for oocyte developmental competence.

Limited work has been performed on embryo PR expression however there were varied, contradictory results. Expression of PR during the preimplantation stages of pig and mouse embryos has been shown (48, 49, 51-54, 56, 57). P4 receptor mRNA was present during all stages of bovine embryo development (51). PR mRNA and protein were expressed in pre-implantation pig embryos prior to the fifth cell division but not at later stages through blastocyst formation. P4 receptor mRNA was undetectable until the blastocyst stage (54).

We have located no studies on PR expression during early organogenesis. However, mRNA and protein are expressed in increasing amounts in the female reproductive tract of the rat after organogenesis (58). In the female reproductive system, PR is expressed in the uterus, mammary gland, ovarian tissue, fallopian tubes (57) and placenta (59).

### P4 and oocyte maturation

Resumption of meiosis in oocytes is triggered by steroid hormones, specifically P4, in certain species (60). The resumption of meiosis and its progression to MII in several mammalian species such as cows, sheep and pigs is steroid dependent and the inhibition of steroidogenesis in ovine follicles leads to impairment of resumption of meiosis and progression to MII. According to research, levels of P4in follicular fluid and its ratio to the estrogen levels are strongly associated with oocyte quality and maturity (61). However, controversy exists regarding the effect of P4 on *in vitro* oocyte maturation (IVM). Our investigations have shown that addition of P4 (10, 38, 50, 100 μM) to the *in vitro* maturation media of mouse GV oocytes could not improve maturation rates and developmental competence of GV in COCs and cumulus denuded oocytes (CDOs) at any of the tested concentrations when compared to the control groups. When we increased P4 from 10 to 100 μM in the culture medium, the maturation rate decreased in a dose dependent manner and the GV arrested rates increased. Research has shown that the effect of P4 in inhibition of meiotic resumption was more effective in COC than CDO (62). It seems there were intensive interactions between oocytes and the surrounding cumulus cells. Oocytes could affect cumulus cell functions.

Vanderhyden et al. (63) have shown that mouse oocytes modulate steroid production by the surrounding cumulus cells. These observations have suggested that oocytes secret a factor (s) which control cumulus cell production of E2 and P4. In contrast, Jamnongjit et al. (64) observed that testosterone or P4 and epidermal growth factor induced meiotic resumption in mouse oocytes during their *in vitro* maturation; the effect of these steroids could have been inhibited by specific receptor antagonists. Fukui et al. (65) demonstrated that P4 supplementation of IVM culture systems decreased the rate of bovine oocyte maturation and that addition of P4 to fertilization culture medium did not improve the number of cleavage stage embryos. Carter et al. (66) have shown that addition of P4 to culture medium did not affect the proportion of *in vitro* matured/*in vitro* fertilized zygotes that developed to the blastocyst stage *in vitro*. There was no effect on conceptus elongation following transfer to synchronized recipient heifers.

Elsewhere, the role of P4 on bovine oocyte developmental competence has been investigated by inhibiting P4 production of cumulus cells. Research has shown that supplementation of oocyte maturation medium with trilostane, an inhibitor of 3 β-hydroxy steroid dehydrogenase, caused a significant decrease in the blastocyst formation rate, which was completely reversed by the addition of P4 or a P4 agonist. This observation might support a positive rolefor P4 in oocyte quality (39).

Supplementation of canine oocyte culture media with steroid hormones stimulates their nuclear maturation (67, 68). However, this effect of steroid hormones has not been shown in anestrus bitches (69). In rhesus monkeys, the improvement of in vitro oocyte development was demonstrated in the presence of P4 and E2 (68).

Overall, these inconsistent results may be due to different experimental strategies that have been used. It seems that the length of time between LH or human chorionic gonadotropin (hCG) stimulation and GV breakdown (GVBD) might explain differences among mammalian species. Possibly, maintaining the healthiness of the oocyte and its ability to mature in species with a long dormant period between LH surge and GVBD requires steroid support.

It has been shown that the P4 antagonist mifepristone (RU486) which occupies PR could not reverse the inhibitory effect of P4 on oocyte maturation (62). Therefore, it has been concluded that the inhibition of mouse oocyte maturation by P4 is not receptor dependent. It appears that P4 could inhibit cAMP phosphodiesterase (PDE) activity through binding to the purine-binding site of this enzyme, which in turn inhibits meiosis by increasing oocyte cAMP levels (70, 71).

### P4 and oocyte fertilization

The role of steroids has been shown to be involved in the acquirement of meiotic competence and the ability to undergo normal fertilization and development to the blastocyst stage (33). In humans and rhesus monkeys, high ratios of P4 to E2 in follicular fluid were associated with better embryo development (72).

In this regard we attempted to investigate the effect of P4 in concentrations similar to that of preovulatory follicular fluid (10 and 38 μM) on developmental competence of mouse GV oocytes and subsequent fertilization potential. Our experiments showed that P4 could not increase the fertilization rate and development of the embryo to the blastocyst stage (62). The result of this experiment was inconsistent with other studies (73-75). Silva and Knight (76) have shown that the addition of P4 to bovine oocyte *in vitro* maturation medium reduced the rate of blastocyst formation.

Mattioli et al. (77) reported that presence ofP4 in porcine oocyte maturation medium increased subsequent sperm head decondensation and male pronuclei formation. Zhang and Armstrong (78) reported that the addition of P4 to porcine oocyte maturation medium could increase both fertilization and cleavage rates, whereas E2 could not. P4 had the opposite effect in ovineoocytes (74).

### In vivo embryo development and P4

The embryo develops in tubal and uterine microenvironments that are mainly controlled by P4. P4 may act directly as a survival factor or indirectly promote the production and secretion of cytokines which contribute to embryonic survival and development (79). It has been shown that P4 elevation occurs when the embryo does not reach the uterus, thus this finding proposes that the effect of P4 on embryo development is mediated via P4-induced changes in the endometrial transcriptome (80).

Granulocyte macrophage colony stimulating factor is a cytokine secreted by the embryo and endometrium under control of P4 (81) which promotes embryo development (82). Lessey et al. (83) have shown an increase in growth factor production in the stromal cells in response to P4 administration.

Low P4 levels have been linked to early pregnancy failure (84) and poor embryo development (85), while in cattle administration of P4 enhances conceptus development (66, 86).

Some studies have shown that endogenous P4 is a main factor in the preparation of the endometrium for embryo implantation (87, 88). Due to the effects of P4 on improving pregnancy rates of IVF patients in the ART clinic, thus exogenous P4has been used as luteal support to enhance implantation rates (89). Although P4 is essential for continuation of pregnancy in all mammals, expressions of P4 receptors cease prior to implantation. It seems the loss of P4 receptors is important for maternal recognition and embryo development in early pregnancy (90).

Several studies have described the effect of exogenous P4 supplementation on embryo development with varying results, according to the time and duration of P4 treatment (91-95).

Initiation of P4 supplementation at the time of onset of the postovulatory rise (between days 4 and 5) resulted in consistent increases in pregnancy rate, however when P4 was administered later there was no improvement in pregnancy rate (92). The results of another study have suggested that the time of/or strength of the postovulatory P4 rise is critical for embryo development rather than the final concentration of P4in the luteal phase (91). Use of supraphysiological levels of P4 during early pregnancy in the mouse has resulted in a similar conclusion (96).

In our previous study we compared embryo quality and implantation rate in pregnant mice in superovulated, P4 treated and superovulated- P4 treated groups. Our observation showed a high survival rate of blastocysts (97.68%) and implantation rate (92.06%, p< 0.001) in pregnant mice from the P4-treated group compared to the P4 superovulated group, which meant that injections of 1 mg/mouse of progesterone in un-stimulated mice significantly improved implantation rates compared to the control and super ovulated groups (28).

### *In vitro* embryo development and P4

To answer the question of whether P4 directly affects embryo development or there is an indirect effect via changes in the endometrium, some researchers have added P4 to embryo culture medium *in vitro* and examined development to the blastocyst stage, with contradictory results (2, 97-100). These contradictory observations might be attributed to different culture systems which have been used.

*In vitro* and in vivo experiments by Clemente et al. (57) showed that the effects of P4 on conceptus elongation could be due to a direct effect of P4 on the embryo. They demonstrated that a P4 receptorwas expressed in all stages of embryo development. These researchers showed the direct effect of P4 on embryo development. Supplementation of simple or co-culture embryo culture systems with P4 did not affect on the embryo development and blastocyst cell number. However, *in vitro*-derived embryo transfer to a recipient treated with P4 resulted in a four-fold increase in conceptus length on day 14. These data confirmed the hypothesis that conceptus elongation in cattle was related to P4- induced changes in the uterine environment (57). This finding agreed with a study by Geisert et al. (101) who showed that administration of P4 early in the estrous cycle advanced uterine receptivity for the transfer of older asynchronous embryos. Supplementation of embryo culture medium with lipidsoluble P4 resulted in an increase in the numbers of embryos that developed to the blastocyst stage (28, 99, 102-106). However different observations were reported by other studies (76, 99, 107).

Ferguson et al. (97) have demonstrated that addition of physiological concentrations of P4 to embryo culture medium at three days post-insemination benefitted embryo development in several ways. Thus, they have concluded that P4 has a direct positive effect on the developing *in vitro* culture of bovine embryos. P4 supplementation increased the number of *in vitro* culture embryos that developed to the grade 1 blastocyst stageas well as the number of hatched blastocysts.

It was shown that co-culture of an embryo with endometrial tissue cultured in the presence of P4 and E2 benefitted embryo development (108).

Also, our investigations led to similar results in which *in vitro* culture of mouse 2-cell embryos in the presence of 20 ng/ml P4 resulted in a high proportion of embryos that reached the blastocyst stage. In this study,embryo quality was less affected by P4 (28).

## Conclusion

These results have shown that P4 could be a factor for embryonic survival and improve in vivo embryo development and implantation, both directly and indirectly. on its concentration and the mammalian species. The effect of P4 on oocyte maturation and embryo development may be dependent on its concentration and the mammalian species.
